# Enantiospecific antitrypanosomal *in vitro* activity of eflornithine

**DOI:** 10.1371/journal.pntd.0009583

**Published:** 2021-07-12

**Authors:** Mikael Boberg, Monica Cal, Marcel Kaiser, Rasmus Jansson-Löfmark, Pascal Mäser, Michael Ashton

**Affiliations:** 1 Unit for Pharmacokinetics and Drug Metabolism, Sahlgrenska Academy, University of Gothenburg, Gothenburg, Sweden; 2 Parasite Chemotherapy Unit, Department of Medical Parasitology and Infection Biology, Swiss Tropical and Public Health Institute, Basel, Switzerland; 3 University of Basel, Basel, Switzerland; 4 DMPK, Research and Early Development Cardiovascular, Renal and Metabolism, BioPharmaceuticals R&D, AstraZeneca, Gothenburg, Sweden; University of Texas Southwestern Medical School, UNITED STATES

## Abstract

The polyamine synthesis inhibitor eflornithine is a recommended treatment for the neglected tropical disease Gambian human African trypanosomiasis in late stage. This parasitic disease, transmitted by the tsetse fly, is lethal unless treated. Eflornithine is administered by repeated intravenous infusions as a racemic mixture of L-eflornithine and D-eflornithine. The study compared the *in vitro* antitrypanosomal activity of the two enantiomers with the racemic mixture against three *Trypanosoma brucei gambiense* strains. Antitrypanosomal *in vitro* activity at varying drug concentrations was analysed by non-linear mixed effects modelling. For all three strains, L-eflornithine was more potent than D-eflornithine. Estimated 50% inhibitory concentrations of the three strains combined were 9.1 μM (95% confidence interval [8.1; 10]), 5.5 μM [4.5; 6.6], and 50 μM [42; 57] for racemic eflornithine, L-eflornithine and D-eflornithine, respectively. The higher *in vitro* potency of L-eflornithine warrants further studies to assess its potential for improving the treatment of late-stage Gambian human African trypanosomiasis.

## Author summary

The neglected tropical disease human African trypanosomiasis is lethal unless treated. One of the treatments for the late stage–i.e. when parasites have invaded the central nervous system–of Gambian human African trypanosomiasis is the drug eflornithine, which is dosed as 50:50 racemic mixture of the two enantiomers L-eflornithine and D-eflornithine. This study showed that L-eflornithine was better than D-eflornithine at inhibiting the growth of parasites *in vitro*. The 50% inhibitory concentration for L-eflornithine was 5.5 μM in comparison to 50 μM for D-eflornithine. This higher *in vitro* potency for L-eflornithine warrants further studies to assess its potential as an improved treatment for late-stage Gambian human African trypanosomiasis.

## Introduction

The neglected tropical disease human African trypanosomiasis (HAT), also known as sleeping sickness, is fatal unless treated. The amino acid analogue DL-alpha-difluoromethylornithine, known as eflornithine, was first developed for oncological use [1] and later discovered to have antitrypanosomal activity [2]. Eflornithine, included in the World Health Organization (WHO) model list of essential medicines [3], is dosed intravenously, commonly together with oral nifurtimox, to treat the late stage of Gambian HAT [4–7], which account for 98% of the total HAT cases [8]. The intravenous administration of eflornithine requires hospital-like settings. Treatment accessibility in rural areas would increase if an oral eflornithine treatment was available with easier and less costly logistics [9]. However, clinical trials with oral racemic eflornithine have failed to achieve sufficiently high systemic exposure, most likely due to poor bioavailability at maximum tolerated oral dose [9,10]. The two enantiomers, L- and D-eflornithine, both inhibited the target enzyme ornithine decarboxylase (ODC) in a cell free assay with human ODC [11]. However, the potential difference in antitrypanosomal efficacy on a parasite level may limit the possibility for oral treatment since the enantiomers differ in their oral bioavailability [12]. This study aimed to investigate the antitrypanosomal *in vitro* activities of racemic eflornithine, L-eflornithine and D-eflornithine against three *Trypanosoma brucei* (*T*.*b*.) *gambiense* strains to support whether a future late-stage Gambian HAT treatment with a potentially more active enantiomer would be feasible or not.

## Materials and methods

### Compounds

Eflornithine hydrochloride was donated by the UNICEF/UNDP/World Bank/WHO Special Programme for Research and Training in Tropical Disease ([TDR], Geneva, Switzerland). L-eflornithine and D-eflornithine were separated from the racemic mixture by semi-preparative liquid chromatography [13]. Racemic eflornithine, L-eflornithine and D-eflornithine were dissolved in sterile water for the *in vitro* activity assay and diluted in culture medium before incubation of *T*.*b*. *gambiense* parasites in 96-well plates.

### Parasites and cell culture conditions

The *T*.*b*. *gambiense* strain STIB930 is a derivative of the strain TH1/78E (031), which was isolated in 1978 from a patient in Côte d’Ivoire [14]. The K03048 strain was isolated from a patient in South Sudan in 2003 [15]. The 130R strain was isolated 2003 from a patient in the Democratic Republic of the Congo [16]. Parasite incubation conditions were 37°C, 5% CO_2_ atmosphere, in HMI-9 medium [17] with fetal bovine serum and human serum, 15% and 5%, respectively. Parasites were subcultured at appropriate dilutions every two to three days to ensure maintenance in exponential growth phase.

### *In vitro* growth inhibition assays

Racemic eflornithine, L-eflornithine and D-eflornithine were tested in an AlamarBlue serial drug dilution assay, described in detail elsewhere [18], in order to quantify parasite growth inhibition. In brief, serial drug dilutions were prepared in 96-well microtiter plates containing HMI-9 medium. Pre-experimental parasites counts were obtained using a CASY cell counter (OLS OMNI Life Science, Bremen, Germany) before the wells were inoculated with 100,000 *T*.*b*. *gambiense* parasites and incubated for 72 hours. The fluorescent agent resazurin was added before the plates were incubated for another four to six hours. SpectraMax Gemini XS microplate fluorescence scanner was used to read the plates at the excitation and emission wavelengths 536 nm and 588 nm, respectively. To determine the *in vitro* growth inhibition, the study was conducted with five independent experiments for the STIB930 *T*.*b*. *gambiense* strain with racemic eflornithine and seven with L-eflornithine or D-eflornithine, respectively. Four independent experiments were performed for the K03048 and 130R *T*.*b*. *gambiense* strains with racemic eflornithine and six with L-eflornithine or D-eflornithine, respectively. Time-dependence for the drug exposure was studied for racemic eflornithine, L-eflornithine and D-eflornithine in a series of *in vitro* growth inhibition assays where the *T*.*b*. *gambiense* strain STIB930 was under drug exposure for 24, 48 or 72 hours. All other parts of the experiment followed a similar protocol as the AlamarBlue serial drug dilution assay and plate readings as previously described. Racemic eflornithine, L-eflornithine and D-eflornithine were tested in an *in vitro* cytotoxicity assay with L6 rat skeletal myoblast cells using a protocol described in full elsewhere [19]. The positive control in the cytotoxicity *in vitro* assay was podophyllotoxin with a known 50% inhibitory concentration (IC_50_) of 0.02 μM (0.007 μg/mL).

### Data and statistical analyses

Eq 1 was fitted to the antitrypanosomal *in vitro* activity data using non-linear mixed effects modelling as implemented in Phoenix software (Version 8.2, Certara, Princeton, NJ, USA). Firstly, each combination of compound and parasite strain was fitted separately by naïve pooled data analysis to estimate IC_50_, sigmoidicity factor gamma (γ) that characterizes the concentration-inhibition relationship steepness and maximum inhibition (I_max_) where I_0_ represents the baseline effect without drug exposure according to:

$$Inhibition=I_{0}-\frac{I_{max}\times {Concentration}^{\gamma }}{{IC}_{50}^{\gamma }+{Concentration}^{\gamma }}$$ (1)

In a second step, each compound was separately fitted to pooled data for all strains. For model validation, parameter estimate plausibility was assessed and bootstrap (n = 1000) using the first-order conditional estimate-extended least square method was performed. The bootstrap estimates were used to establish the 5^th^ and 95^th^ percentiles for the model predictions. Differences in parameter estimates from the bootstrap were assessed as statistically significant for 95% confidence intervals (95% CI) without overlap. For discrimination between nested models with γ = 1 or estimated γ in non-linear mixed effects modelling, a decrease in -2 log likelihood over 3.84 for the more complex model was regarded as statistically significant (P < 0.05) with an assumed χ^2^ distribution for the difference in -2 log likelihood. Plots and statistical analysis were made using Rstudio (Version 1.3.1093) with the R software (Version 4.0.3, 2020, The R foundation for Statistical Computing).

## Results

### Antitrypanosomal *in vitro* activity against STIB930, K03048 and 130R

**Fig 1:**
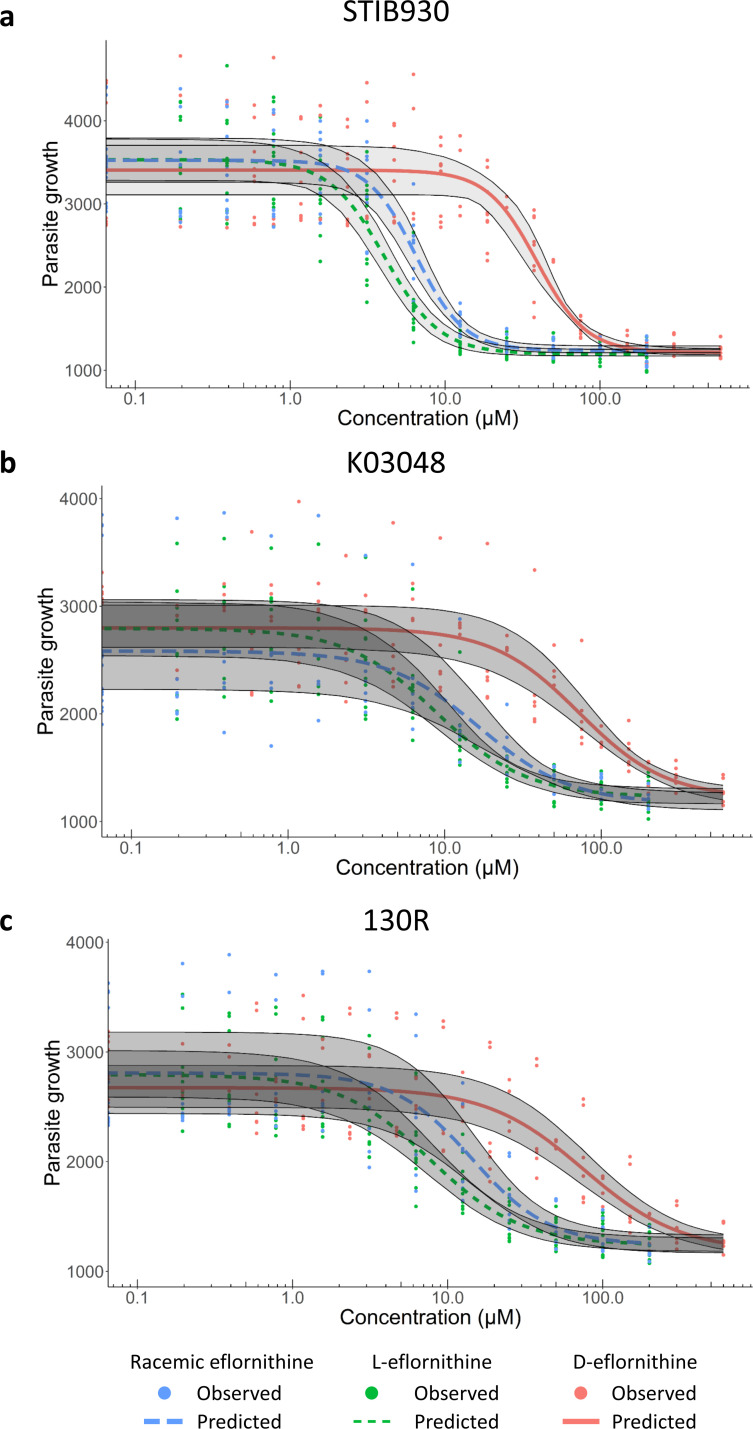
Antitrypanosomal *in vitro* activity for racemic eflornithine and its enantiomers against three different *T*.*b*. *gambiense* strains. *In vitro* activity for racemic eflornithine (blue), L-eflornithine (green) and D-eflornithine (red) against *T*.*b*. *gambiense* strains a) STIB930, b) K03048 and c) 130R. Parasite growth values are shown as relative fluorescence in the AlamarBlue serial drug dilution assay. Dots represent observed experimental data, lines the model predictions and grey areas the 5^th^ to 95^th^ percentiles of the model prediction central values.

All compounds inhibited the growth of the three *T*.*b*. *gambiense* strains in a concentration-dependent manner (Fig 1). L-eflornithine had the lowest IC_50_ estimates throughout, with 4.1 μM (95% CI 3.1; 5.0), 8.9 μM (7.0; 11) and 7.7 μM (6.8; 8.5) for strains STIB930, K03048 and 130R, respectively. D-eflornithine was less potent with IC_50_ estimates of 39 μM (29; 49), 73 μM (62; 85) and 76 μM (66; 86) for the same strains. IC_50_ values for racemic eflornithine were 6.4 μM (5.2; 7.7), 17 μM (15; 18) and 14 μM (12; 17) for STIB930, K03048 and 130R, respectively (Table 1).

**Table 1:** IC_50_, gamma and I_max_ estimates for racemic eflornithine, L-eflornithine and D-eflornithine in three different *T*.*b*. *gambiense* strains and overall across all strains.

Parameter	Drug	STIB930 Estimate (95% CI)	K03048 Estimate (95% CI)	130R Estimate (95% CI)	Overall Estimate (95% CI)
**IC**_**50**_ **(μM)**	Racemic eflornithine	6.4 (5.2 to 7.7)	17 (15 to 18)	14 (12 to 17)	9.1 (8.1 to 10)
	L-eflornithine	4.1 (3.1 to 5.0)	8.9 (7.0 to 11)	7.7 (6.8 to 8.5)	5.5 (4.5 to 6.6)
	D-eflornithine	39 (29 to 49)	73 (62 to 85)	76 (66 to 86)	50 (42 to 57)
**Gamma**	Racemic eflornithine	2.8 (2.4 to 3.4)	1.5 (1.3 to 1.6)	1.7 (1.4 to 1.9)	1.7 (1.5 to 2.1)
	L-eflornithine	2.5 (1.9 to 3.2)	1.5 (1.3 to 1.7)	1.5 (1.4 to 1.6)	1.6 (1.3 to 1.8)
	D-eflornithine	2.8 (2.0 to 4.0)	1.6 (1.2 to 1.8)	1.4 (1.1 to 1.7)	1.7 (1.3 to 2.2)
**I**_**max**_	Racemic eflornithine	0.95 (0.92 to 0.97)	0.93 (0.88 to 0.98)	0.94 (0.91 to 1.0)	0.94 (0.91 to 0.98)
	L-eflornithine	0.96 (0.94 to 0.98)	0.92 (0.87 to 0.95)	0.95 (0.92 to 0.98)	0.94 (0.92 to 0.97)
	D-eflornithine	1.0 (0.96 to 1.0)	0.93 (0.88 to 0.99)	0.93 (0.89 to 0.98)	0.97 (0.93 to 1.0)
**Residual variability**	Racemic eflornithine	0.12 (0.090 to 0.14)	0.15 (0.054 to 0.22)	0.15 (0.068 to 0.18)	0.18 (0.15 to 0.19)
	L-eflornithine	0.13 (0.11 to 0.15)	0.13 (0.092 to 0.17)	0.12 (0.082 to 0.14)	0.16 (0.14 to 0.17)
	D-eflornithine	0.14 (0.11 to 0.17)	0.14 (0.089 to 0.18)	0.14 (0.085 to 0.17)	0.17 (0.15 to 0.19)

Parameters were estimated with bootstrap (n = 1000), 95% CI– 95% confidence interval

### Growth inhibition analysis for all strains pooled

**Fig 2:**
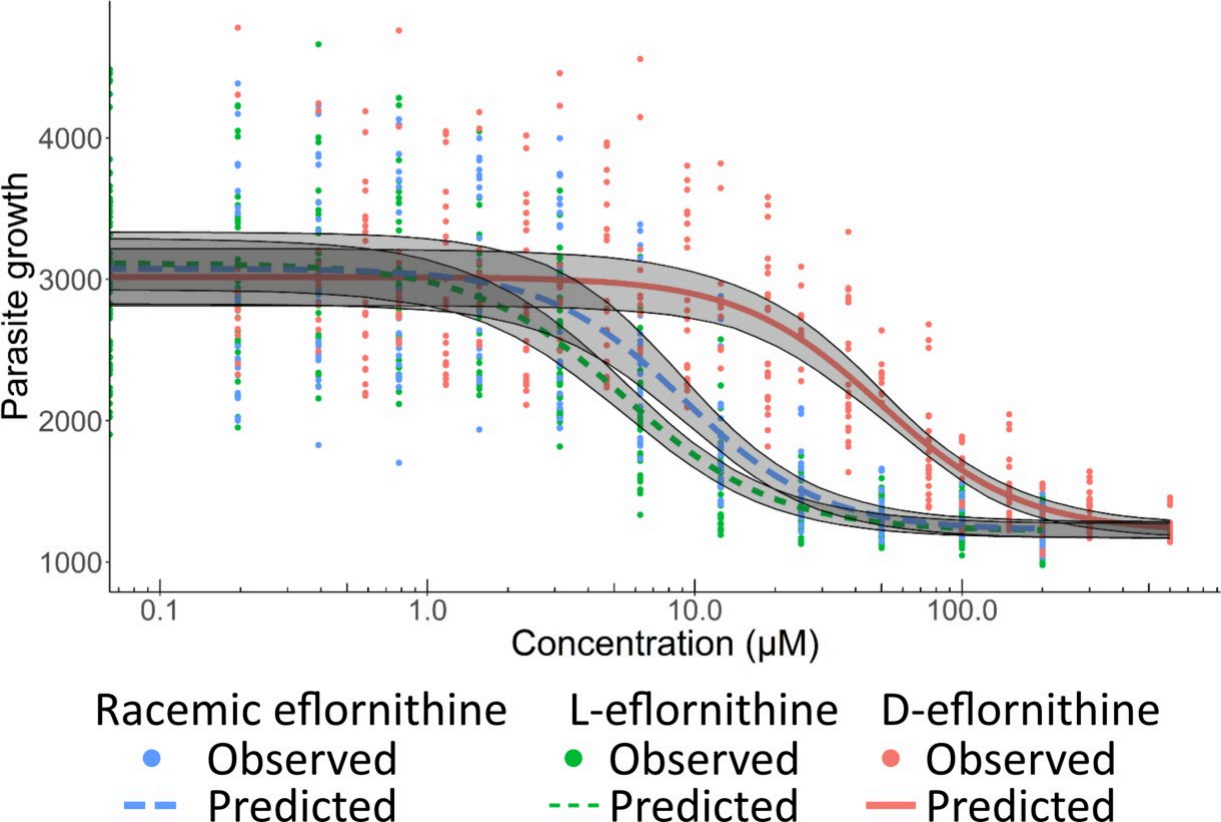
The overall antitrypanosomal *in vitro* activity was elicited by the more active L-eflornithine enantiomer. Antitrypanosomal activity for racemic eflornithine (blue), L-eflornithine (green) and D-eflornithine (red) against three *T*.*b*. *gambiense* strains collectively. Parasite growth values are shown as relative fluorescence in the AlamarBlue serial drug dilution assay. Dots represent observed experimental data, lines the model predictions and grey areas the 5^th^ to 95^th^ percentiles of the model prediction central values.

Pooling the data for the three strains resulted in IC_50_ estimates (95% CI) of 9.1 μM (8.1; 10), 5.5 μM (4.5; 6.6), and 50 μM (42; 57) for racemic eflornithine, L-eflornithine and D-eflornithine, respectively. The sigmoidicity factor γ and I_max_ values were similar for racemic eflornithine, L-eflornithine and D-eflornithine (Table 1). The 5^th^ to 95^th^ percentiles for model predictions did not overlap at concentrations close to IC_50_ values for the three treatments (Fig 2). The overall *in vitro* 90% inhibitory concentration (IC_90_) for racemic eflornithine, L-eflornithine and D-eflornithine were 25 μM, 17 μM and 166 μM, respectively. The growth inhibition was time-dependent as the concentration antitrypanosomal *in vitro* activity relationship after 72 h was steeper, and with a lower IC_50_ estimate compared to the IC_50_ estimates for 24 h and 48 h drug exposure times (S1 Fig and S1 Table). No observations of *in vitro* cytotoxicity were made in the L6 cell assay at relevant *in vitro* concentrations for racemic eflornithine, L-eflornithine and D-eflornithine whereas the positive control podophyllotoxin was cytotoxic with an expected IC_50_ at approximately 0.02 μM (S2 Fig).

## Discussion

The enantiospecific eflornithine antitrypanosomal activity is to the best of our knowledge documented herein for the first time. The overall *in vitro* potency of L-eflornithine was about 9-fold higher than D-eflornithine against three *T*.*b*. *gambiense* strains. As a result, the IC_50_ estimate for racemic eflornithine was approximately twice that of L-eflornithine due to 1:1 inclusions of much less potent D-eflornithine. The difference in antitrypanosomal activity could possibly be due to an enantioselective eflornithine transport into the *T*.*b*. *gambiense* parasites since the enantiomers appear to have similar inactivation properties on an enzyme level [11]. Clinically, total eflornithine concentrations in cerebrospinal fluid over 50 μM, equating to approximately 5.5 times the overall *in vitro* IC_50_ in the present study, have been associated with efficient parasite eradication in late-stage Gambian HAT patients after intravenous infusions of racemic eflornithine [9,20]. The higher potency for L-eflornithine observed in the present study suggests that this threshold value could potentially be decreased by approximately 50% if pure L-eflornithine were administered. Supporting this hypothesis, cerebrospinal fluid concentrations over 23 μM for L-eflornithine, equating to approximately 4 times the overall *in vitro* IC_50_ in the present study, were associated, however not statistically significant, with probability of cure in a clinical study of 25 patients when treated with racemic eflornithine orally [12]. A prospective clinical trial investigating the clinical efficacy of L-eflornithine dosed intravenously and orally at appropriate, tolerated doses could elucidate the clinical potential for L-eflornithine. The pharmacological effect of eflornithine in late-stage Gambian HAT may be expected to depend predominantly on unbound L-eflornithine concentration in the systemic circulation and central nervous system. The plasma protein binding for racemic eflornithine has been reported as negligible [21]. Total eflornithine concentrations are in such case expected to be identical to unbound concentrations and available to target the *T*.*b*. *gambiense* parasites. The IC_50_ values for the antitrypanosomal *in vitro* activity in the present study could therefore, with more confidence, be translated to *in vivo* relevant concentrations. The pharmacodynamic effect and cure can be seen as conditioned by critical interactions between the drug, the patient and the *T*.*b*. *gambiense* parasite as discussed for other antimicrobial agents [22].

In a more pharmacological and dose-finding oriented perspective, as discussed for antimalarial treatments, the *in vitro* IC_90_ can be used as a free drug minimum inhibitory concentration surrogate [23]. This approach has been successful when, for instance, translating *in vitro* findings to clinically relevant minimum inhibitory concentration proxy [24]. For Gambian HAT, the IC_90_ values in the present study for L-eflornithine and racemic eflornithine at 17 and 25 μM, respectively, were exceeded in serum and cerebrospinal fluid after fourteen days of racemic eflornithine treatment with two-hour intravenous infusions at 100 mg/kg four times per day [25]. Currently, the clinical posology for racemic eflornithine is 200 mg/kg twice daily when combined with nifurtimox [26]. Extrapolation of *in vitro* IC_50_ or IC_90_ to *in vivo* relevant values of efficacious unbound drug concentration in plasma may be fraught with error since effects also depend on whether the drug reaches its target tissue and on the role of the immune system *in vivo* [27]. Uptake of eflornithine into the central nervous system is low leading to a poor partitioning between plasma and brain or cerebrospinal fluid [28,29]. The reported clinical cerebrospinal fluid to plasma or serum ratios range from 0.1 to 0.5 [9,12,25]. Eflornithine partitioning from plasma to cerebrospinal fluid appears to be non-stereoselective when administered as a racemate orally [12]. Additionally, it is important to take the factors of target occupancy, target turnover and active metabolites into account in *in vitro*–*in vivo* extrapolation. For eflornithine, no metabolites have been identified, hence can not contribute to pharmacological effects [30]. Moreover, since eflornithine can be seen as a slow acting compound [21], and trypanostatic rather than trypanocidal [31], the pharmacokinetic/pharmacodynamic relationship is important to consider as drug transporters in the body and/or *T*.*b*. *gambiense* parasites involved in the drug disposition could affect the clinical efficacy of eflornithine.

Only three *T*.*b*. *gambiense* strains were tested in the study which is a limitation. Granted, an analysis with more strains would render more generalizable approximations when extrapolating from the *in vitro* results to the clinic. Eflornithine resistance has been associated with non-expression of the *TbAAT6* transporter gene [32]. This TbAAT6-dependent eflornithine transport into *T*.*b*. *gambiense* parasites has been investigated further where lines of trypanosomes showed lower sensitivity to eflornithine when the *TbAAT6* transporter gene was silenced [33]. If the uptake by this amino acid transporter disfavours D-eflornithine, it might contribute to the observed higher *in vitro* activity for L-eflornithine in the present study. Radiolabelled compound could be used to decouple the potentially enantioselective transport of eflornithine into *T*.*b*. *gambiense* parasites. *In vivo* studies with L-eflornithine would potentially increase the confidence in the presented findings; however, the experiments mentioned above were assessed as outside of the study scope.

To achieve and sustain global elimination of HAT [34], it is imperative to design, make, test and analyse results for novel compounds in the pipeline. For both patients and care givers, an oral route of administration of drugs would be much preferred. Oral administration of racemic eflornithine has been investigated in clinical [9,20,21,25,35–38] and preclinical [12,39,40] studies but the antitrypanosomal efficacy and tolerability of enantiopure L-eflornithine is still to be investigated. The mechanisms and the potential enantioselectivity of the noted gastrointestinal side effects in the clinical studies with oral racemic eflornithine remain so far unknown. An oral alternative HAT treatment, fexinidazole, has been approved [41,42] and is first line treatment for patients with a cerebrospinal fluid leucocyte count less than 100 per μL. Acoziborole is currently in clinical trials [43]. Overall, these advances are important to achieve global elimination of HAT.

In conclusion, the present study showed that the L-eflornithine enantiomer elicited higher antitrypanosomal *in vitro* activity, as it was more effective than D-eflornithine against three different *T*. *b*. *gambiense* strains *in vitro*. This knowledge could be used in the future to predict *in vivo* efficacious doses of the more active L-eflornithine enantiomer using pharmacokinetic/pharmacodynamic models to assess the feasibility of L-eflornithine treatment for late-stage Gambian HAT.

## Supporting information

S1 Fig Time-dependent antitrypanosomal *in vitro* activity for eflornithine and its enantiomers. Time-dependent *in vitro* activity for a) racemic eflornithine (blue dashed lines), L-eflornithine (green small dashed lines) and D-eflornithine (red full lines) after 24 h (thick lines), 48 h (medium lines) and 72 h (thin lines) of drug exposure. Parasite growth values are shown as relative fluorescence in the AlamarBlue serial drug dilution assay. Dots represent observed experimental data and lines the model predictions. b) Mean IC_50_ values with error bars showing the standard error of the estimates for racemic eflornithine (blue dashed line), L-eflornithine (green dotted line) and D-eflornithine (red full line) after different drug exposure times. Please note the log_10_ scale on the y-axis in S1b Fig.
(TIFF)

S2 Fig *In vitro* cytotoxicity assay for racemic eflornithine, its enantiomers and podophyllotoxin. a) *In vitro* activity against L6 cells for racemic eflornithine (blue), L-eflornithine (green) and D-eflornithine (red) and b) *in vitro* activity for the positive control podophyllotoxin (dark blue). L6 cell growth values are shown as relative fluorescence in the assay. Dots represent observed experimental data and the coloured lines the model predictions.
(TIFF)

S1 Table IC_50_, gamma, I_max_ and residual variability estimates in the time-dependent assay for racemic eflornithine, L-eflornithine and D-eflornithine. (DOCX)
