# Supplementary figures and images for: Enantiospecific antitrypanosomal in vitro activity of eflornithine
Source: PLoS Negl Trop Dis. 2021 Jul 12;15(7):e0009583. doi: 10.1371/journal.pntd.0009583 (PMC8297939; doi:10.1371/journal.pntd.0009583)

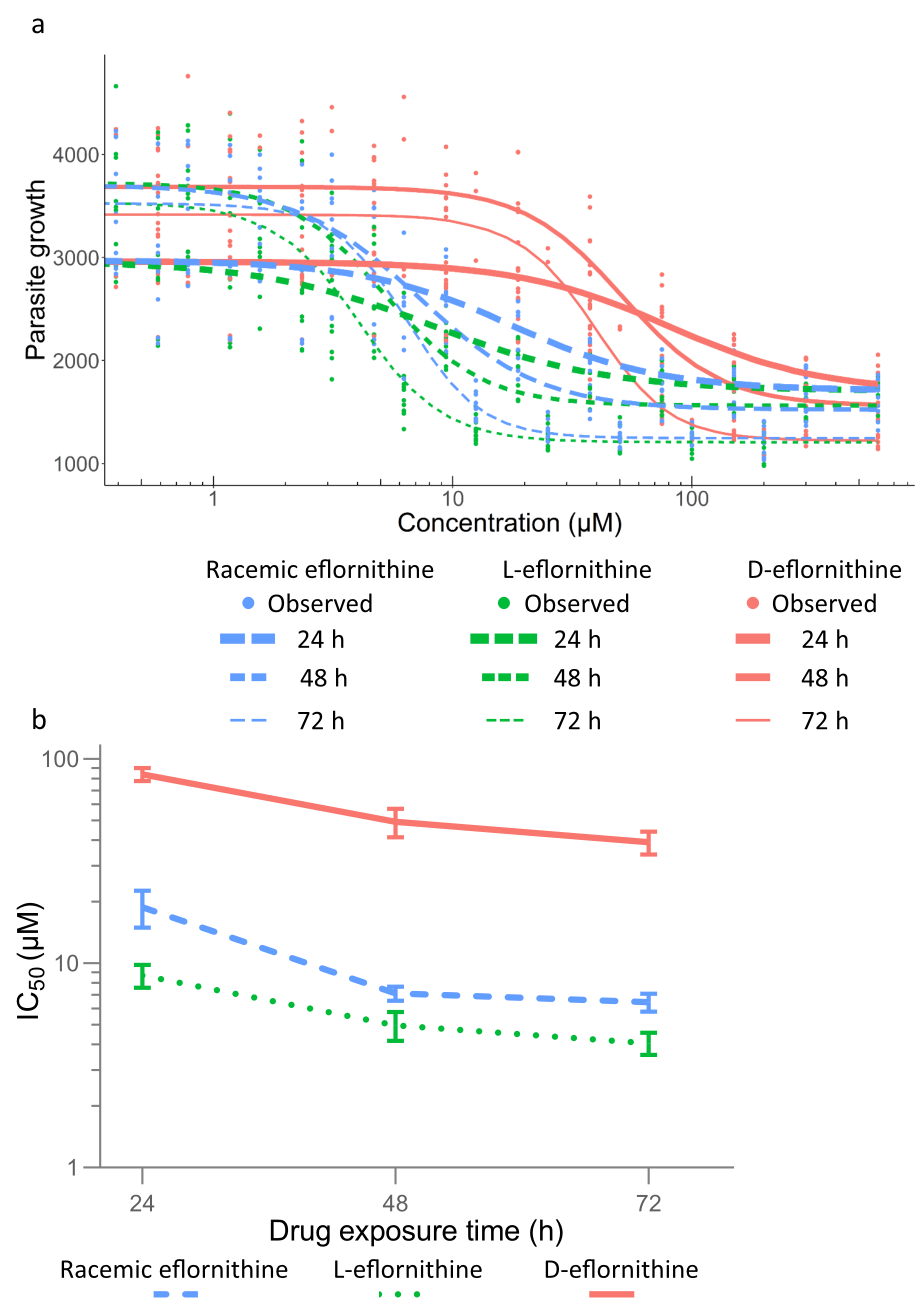

Supplement: S1 Fig — Time-dependent in vitro activity for a) racemic eflornithine (blue dashed lines), L-eflornithine (green small dashed lines) and D-eflornithine (red full lines) after 24 h (thick lines), 48 h (medium lines) and 72 h (thin lines) of drug exposure. Parasite growth values are shown as relative fluorescence in the AlamarBlue serial drug dilution assay. Dots represent observed experimental data and lines the model predictions. b) Mean IC50 values with error bars showing the standard error of the estimates for racemic eflornithine (blue dashed line), L-eflornithine (green dotted line) and D-eflornithine (red full line) after different drug exposure times. Please note the log10 scale on the y-axis in S1b Fig. (TIFF) [file pntd.0009583.s001.tiff]

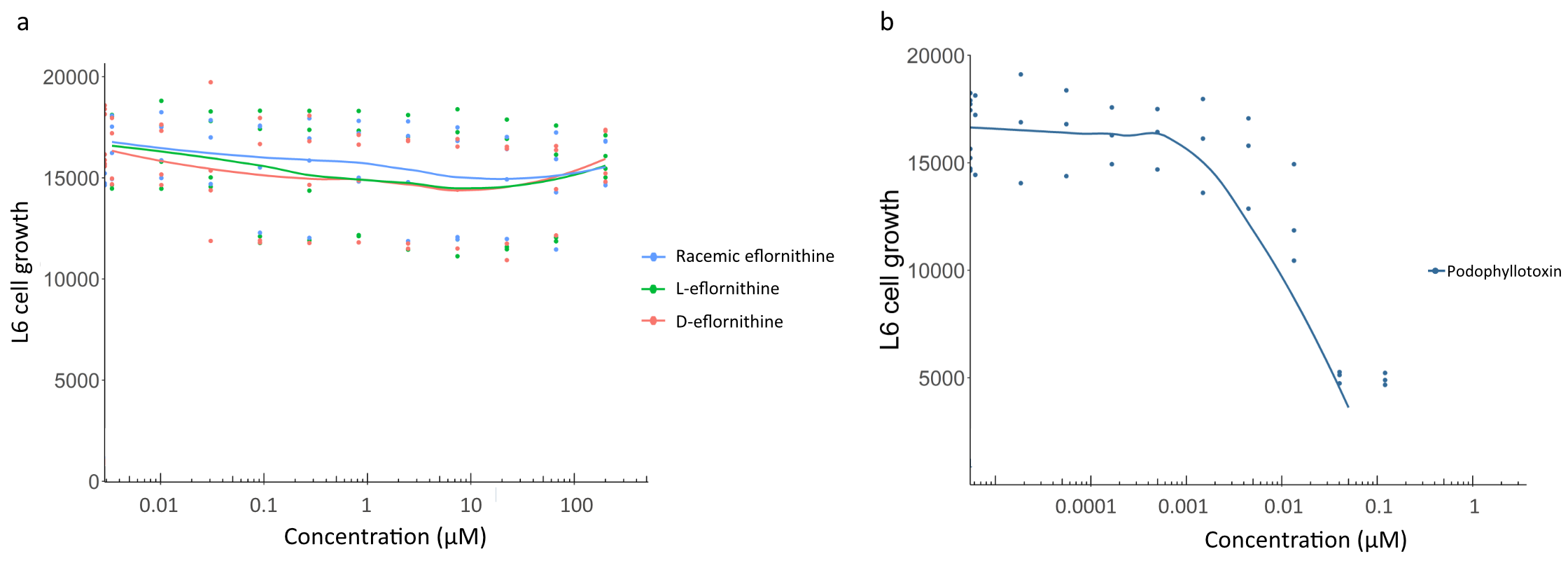

Supplement: S2 Fig — a) In vitro activity against L6 cells for racemic eflornithine (blue), L-eflornithine (green) and D-eflornithine (red) and b) in vitro activity for the positive control podophyllotoxin (dark blue). L6 cell growth values are shown as relative fluorescence in the assay. Dots represent observed experimental data and the coloured lines the model predictions. (TIFF) [file pntd.0009583.s002.tiff]
