# Supplementary material for: Enantiospecific antitrypanosomal in vitro activity of eflornithine
Source: PLoS Negl Trop Dis. 2021 Jul 12;15(7):e0009583. doi: 10.1371/journal.pntd.0009583 (PMC8297939; doi:10.1371/journal.pntd.0009583)
Supplement: S1 Table — (DOCX) [file pntd.0009583.s003.docx]

| **S1 Table. IC_50_, gamma, I_max_ and residual variability estimates in the time-dependent assay for racemic eflornithine, L-eflornithine and D-eflornithine.** | | | | |
| --- | --- | --- | --- | --- |
| **Parameter** | **Drug** | **Incubation time (h)** | **Estimate** | **95% CI** |
| **IC_50_ (µM)** | Racemic eflornithine | 24 | 19 | 15 to 31 |
|  |  | 48 | 7.1 | 5.9 to 8.1 |
|  |  | 72 | 6.4 | 5.2 to 7.6 |
|  | L-eflornithine | 24 | 8.7 | 7.6 to 12 |
|  |  | 48 | 5.0 | 2.8 to 5.9 |
|  |  | 72 | 4.1 | 3.1 to 5.1 |
|  | D-eflornithine | 24 | 84 | 73 to 97 |
|  |  | 48 | 49 | 29 to 60 |
|  |  | 72 | 39 | 30 to 48 |
| **Gamma** | Racemic eflornithine | 24 | 1.4 | 1.2 to 1.5 |
|  |  | 48 | 1.7 | 1.4 to 1.9 |
|  |  | 72 | 2.8 | 2.4 to 3.4 |
|  | L-eflornithine | 24 | 1.2 | 1.0 to 1.4 |
|  |  | 48 | 2.0 | 1.3 to 2.4 |
|  |  | 72 | 2.5 | 1.9 to 3.2 |
|  | D-eflornithine | 24 | 1.4 | 1.3 to 1.5 |
|  |  | 48 | 2.0 | 1.5 to 2.4 |
|  |  | 72 | 2.8 | 2.1 to 4.0 |
| **I_max_** | Racemic eflornithine | 24 | 0.82 | 0.68 to 0.92 |
|  |  | 48 | 0.93 | 0.88 to 0.97 |
|  |  | 72 | 0.95 | 0.92 to 0.98 |
|  | L-eflornithine | 24 | 0.82 | 0.67 to 0.92 |
|  |  | 48 | 0.92 | 0.87 to 0.94 |
|  |  | 72 | 0.96 | 0.94 to 0.98 |
|  | D-eflornithine | 24 | 0.82 | 0.60 to 0.96 |
|  |  | 48 | 0.92 | 0.86 to 0.95 |
|  |  | 72 | 1.0 | 0.96 to 1.0 |
| **Residual variability** | Racemic eflornithine | 24 | 0.13 | 0.081 to 0.17 |
|  |  | 48 | 0.10 | 0.026 to 0.12 |
|  |  | 72 | 0.11 | 0.090 to 0.14 |
|  | L-eflornithine | 24 | 0.13 | 0.075 to 0.17 |
|  |  | 48 | 0.11 | 0.026 to 0.14 |
|  |  | 72 | 0.13 | 0.11 to 0.15 |
|  | D-eflornithine | 24 | 0.15 | 0.078 to 0.20 |
|  |  | 48 | 0.11 | 0.021 to 0.14 |
|  |  | 72 | 0.14 | 0.11 to 0.17 |
| Parameters were estimated with bootstrap (n=1000), 95% CI – 95% confidence interval | | | | |
